# Relapse and inadvertent tooth movement post orthodontic treatment in individuals with fixed retainers: A review

**DOI:** 10.21142/2523-2754-1003-2022-116

**Published:** 2022-09-28

**Authors:** Alicia Chacón-Moreno, María Jimena Ramírez-Mejía, Ana Carolina Zorrilla-Mattos

**Affiliations:** 1 School of Dentistry, Nacional Federico Villareal University, Lima, Peru. ali_ch3@hotmail.com Universidad Nacional Federico Villarreal School of Dentistry Nacional Federico Villareal University Lima Peru ali_ch3@hotmail.com; 2 School of Dentistry, Peruana de Ciencias Aplicadas University, Lima, Peru. jimena.ramirez93@gmail.com Universidad Peruana de Ciencias Aplicadas School of Dentistry Peruana de Ciencias Aplicadas University Lima Peru jimena.ramirez93@gmail.com; 3 School of Dentistry, Privada Antenor Orrego University, Trujillo, Peru. carolinazmattos@outlook.es Universidad Privada Antenor Orrego School of Dentistry Privada Antenor Orrego University Trujillo Peru carolinazmattos@outlook.es

**Keywords:** fixed retainers, recurrence, unwanted movements, relapse, retainers, retention, stability, retenedores fijos, recurrencia, movimientos no deseados, recidiva, retenedores, retención, estabilidad

## Abstract

Orthodontists must fully inform patients about the implications of orthodontic treatment and the subsequent need for retention. This review provides an update on relapse, unwanted movements and different factors that can cause loss of stability following orthodontic treatment. Since it is difficult to predict which patients will present some degree of loss of stability after treatment, it is important that they be treated as if they have a high potential for relapse. The present review included a bibliographic search in the main sources of scientific review including Medline via PubMed, Scopus and the Cochrane library. The search strategy was carried out until May 5, 2022. Only 34 studies fulfilling the selection criteria. Our results showed that maintaining teeth in the correct position following orthodontic treatment is a great challenge for orthodontists. The etiology of relapse is complex and not yet clearly established. Its origin is attributed to factors such as the time of gingival and periodontal tissue reorganization and changes produced by growth, compromising the stability of the results achieved with orthodontic treatment. The retention phase is necessary after orthodontic treatment to avoid relapse or loss of the occlusion results obtained. However, fixed retainers may induce unwanted tooth movement that may occur despite these retainers being attached and intact. There is currently no consensus among orthodontists regarding the ideal type of wire for fixed containment. We concluded that post-orthodontic treatment relapse is the result of a regression towards the original malocclusion. However, changes in the position of the teeth can also occur, which are considered as unwanted movements and have a multifactorial origin.

## RESUMEN

Los ortodoncistas deben informar completamente a los pacientes sobre las implicaciones del tratamiento de ortodoncia y la subsiguiente necesidad de retención. Esta revisión brinda una actualización sobre las recidivas, los movimientos no deseados y los diferentes factores que pueden causar la pérdida de estabilidad después del tratamiento de ortodoncia. Dado que es difícil predecir qué pacientes presentarán algún grado de pérdida de estabilidad después del tratamiento, es importante que sean tratados como si tuvieran un alto potencial de recaída. La presente revisión incluyó una búsqueda bibliográfica en las principales fuentes de revisión científica, incluyendo Medline vía PubMed, Scopus y la biblioteca Cochrane. La estrategia de búsqueda se llevó a cabo hasta el 5 de mayo de 2022. Solo 34 estudios cumplieron con los criterios de selección. Nuestros resultados mostraron que mantener los dientes en la posición correcta después del tratamiento de ortodoncia es un gran desafío para los ortodoncistas. La etiología de la recaída es compleja y aún no está claramente establecida. Su origen se atribuye a factores como el tiempo de reorganización del tejido gingival y periodontal, y a los cambios producidos por el crecimiento, comprometiendo la estabilidad de los resultados alcanzados con el tratamiento de ortodoncia. La fase de retención es necesaria después del tratamiento de ortodoncia para evitar la recaída o la pérdida de los resultados de oclusión obtenidos. Sin embargo, los retenedores fijos pueden inducir un movimiento dental no deseado que puede ocurrir a pesar de que estos retenedores estén colocados e intactos. Actualmente, no hay consenso entre los ortodoncistas con respecto al tipo de alambre ideal para la contención fija. Concluimos que la recidiva postratamiento de ortodoncia es el resultado de una regresión hacia la maloclusión original. Sin embargo, también pueden ocurrir cambios en la posición de los dientes que se consideran movimientos no deseados y tienen un origen multifactorial.

## INTRODUCTION

Stability in orthodontic treatment is an important concern to both orthodontists as well as patients. Relapse involves the loss of stability of orthodontic results and is defined as the tendency of teeth to return to their previous position prior to treatment. Therefore, to achieve stable orthodontic treatment results, good stable occlusion must be ensured, and sometimes, overcorrection of malocclusions. Nonetheless, some studies have described a trend to relapse despite the latter. The etiology of relapse in orthodontics is complex and unclear and involves several factors that compromise the stability of the results, such as the time of gingival and periodontal tissue reorganization, unstable position of the teeth after orthodontic treatment and changes produced by growth ^1^^-^^4^.

On the other hand, some cases present unwanted dental movements that may be caused by a fixed retainer. These movements should not be considered as relapse since they occur in a different direction from the dental position prior to treatment. A common cause of tooth movement caused by the fixed retainer is wire distortion, which can occur if the wire is active at bonding or is even too flexible and deforms during adaptation. Other reasons for wire deformation include trauma, chewing hard foods, and improper flossing ^4^^-^^7^.

On completion of orthodontic treatment with fixed appliances, there is a retention period aimed at preventing relapse ^1^. Retention strategies include the use of fixed or removable retainers or a combination of both in the upper and lower anterior teeth. If the upper arch is expanded or extractions are included in the treatment, a combination of fixed and removable retainers in the maxilla is a common option ^8^. On the other hand, fixed lingual retainers are more frequently used in the mandibular arch. These have the advantage of minimal need for patient cooperation and are currently the most effective and predictable method of stabilizing tooth position, and are considered the gold standard. Nevertheless, adhesion failure is a common clinical complication that can lead to relapse ^3^^,^^9^.

Interestingly, despite the high reliability of fixed retainers bonded to the lingual surfaces of the lower six anterior teeth, some studies have shown that unexpected tooth movements can occur, which in severe cases requires orthodontic retreatment ^5^^,^^9^. These unexpected movements can be considered as relapse if the teeth return to the original malocclusion or are considered unexpected movements if they go in a different direction from the original position, with both types of movement being of concern for the patient and the orthodontist. Finally, growth induces changes in the alignment of the teeth and occlusal relationships throughout life and can even exert forces that favor relapse even when orthodontic retainers are used ^10^. Therefore, this review aimed to evaluate the presence and causes of relapse, as well as the appearance of unwanted dental movements after orthodontic treatment in individuals with fixed retention.

## METHODOLOGY

### Information sources and search strategy

The present literature review included a bibliographic search in the main sources of scientific review including Medline via PubMed, Scopus and the Cochrane library. The search strategy was carried out until May 5, 2022 and is shown in Table 1. 

**Table 1:** Strategies in the search for scientific articles in the main sources of information.

Source	Search terms
PubMed	(((fixed retainers) OR (lingual retainers)) AND (((relapse) OR (recurrence)) OR (unwanted movement))) NOT (((((rats) OR (experimental study)) OR (in vitro)) OR (dogs)) OR (laboratory study))
Cochrane Library	unwanted movements or dental relapse or fixed orthodontic retainers
Scopus	recurrence OR unwanted AND movements OR relapse OR retainers OR retention OR stability AND fixed AND retainers

### Study selection

**Fig. 1:**
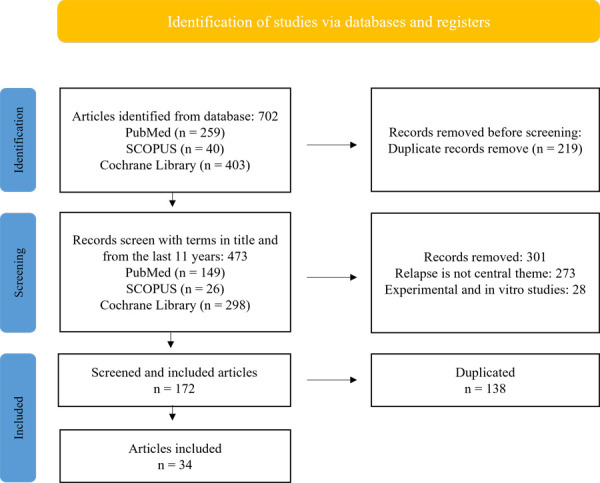
Flow diagram for scientific bibliographic search.

Finally, 34 studies fulfilling the following selection criteria were included: observational studies, randomized clinical trials in human adults of both sexes. *In vitro* studies, literature reviews, letters to the editor, personal opinions and case reports were excluded. (Fig. 1).

## RESULTS AND DISCUSSION

### Orthodontic relapse in individuals using a fixed retainer

For the development of this review, a total of 21 articles were analyzed, of which 14 were selected for their specific contribution of information for the preparation of this analysis.

Maintaining corrected tooth positions after orthodontic treatment is often the most challenging part of orthodontic procedures ^11^^,^^12^. Relapse after orthodontic treatment has traditionally been considered as regression towards the original malocclusion ^13^. However, reversion to the original malocclusion does not always occur, and relapse is considered as any unfavorable change in tooth position after orthodontic treatment ^10^.

Relapse in orthodontics generally occurs as a result of changes related to age due to growth and development or occlusal changes. Teeth stability is also affected by the forces of the periodontal ligament and gingival fibers that surround the teeth and the continuous pressure of the soft tissue structures that tend to return teeth to their anterior positions ^10^^,^^14^.

The retention phase in orthodontics is the final stage of treatment and aims to maintain the teeth in their correct positions after tooth movement is complete ^12^^,^^15^. This has led orthodontists to place retainers to prevent relapse, whether fixed or removable in the post-orthodontic phase, with the objective of achieving permanent retention in order to maintain the results in the long-term after completion of treatment and, thus, prevent relapse ^10^^,^^14^.

Fixed lingual retainers have gained popularity among orthodontics and some studies have shown their effectiveness in preventing relapse in both the long term or permanently following completion of orthodontic treatment ^4^^,^^5^^,^^16^^,^^17^. Fixed retainers may be chosen by orthodontists to control stability caused by changes occurring in the gingival tissues, and also in cases in which changes in the shape of the arch have developed, particularly when the anterior segments are previously displaced or expanded in the mandibular intercanine region ^18^.

There are two types of fixed retainers, one that is attached only to the canines and another that is attached from canine to canine including the incisors ^16^. Patients with fixed retainers bonded only to the canines appear to have a higher rate of incisors irregularity at 5 years after treatment compared to the placement of retention wire also bonded to the incisors. This has led to increased use of bonding to all teeth, although problems may arise following this procedure. Bonding 6 teeth instead of 2 increases the probability of bond failure by 20% to 30% within the first 5 years.^4^

A more serious, albeit very unusual, problem is the involuntary movement of the teeth caused by a lingual retainer. These tooth movements may be unexpected taking into account that the fixed retainers are attached and intact ^4^^,^^5^^,^^16^^,^^17^. Reported failure rates of bonded retainers vary widely between 11% and 53% ^11^^,^^19^^,^^20^. Various causes of unexpected complications in patients with fixed retainers have been suggested, including poor bonding technique. The failure of fixed retention may be due to detachment between the wire and the composite, the rupture of the interface between the adhesive and the enamel, the deformation of the wire ^11^^,^^21^, or insufficient passivity of the wire, the instability of multistrand wires and wire straightening or wire activation by mechanical trauma. Among the complications, torque changes between adjacent mandibular incisors and opposite inclinations of contralateral mandibular canines have also been reported ^11^.

For all these reasons, a successful retention phase depends on many factors, including patient compliance ^20^, making it is essential for patients to understand the implications of orthodontic treatment and the subsequent need for retention ^21^. Although relapse does not occur in all patients, it is clinically difficult to predict which patients will experience changes post-treatment, and therefore, it is important for all patients to be treated as if they have a high potential for relapse ^10^.

### Unwanted dental movements using fixed retainers

In relation to this topic ^10^, specifically selected articles were used. The most serious problems developed following orthodontic treatment are unwanted tooth movements caused by a fixed retainer. These movements are diverse and depend on the wire designs and sizes used ^4^. Movements have been reported to occur in 1.1% to 5% of patients using a fixed retainer cemented to each tooth ^22^.

No fixed retainer is immune to unwanted or unexpected movements and these movements can be measured in the three spatial planes: sagittal (protrusion or retrusion), vertical (extrusive or intrusive), and transverse (clockwise or counterclockwise). In the sagittal plane, the movements are usually protrusive in the incisors and occur in the maxilla, while in the mandible movement is protrusive and especially retrusive in the canines ^5^^,^^9^^,^^11^^,^^22^.

Tooth movements in the vertical plane are extrusive of both arches, being more frequent in the upper arch ^5^^,^^11^. However, another study reported that the lower canines are the most severely affected teeth, presenting significant apicocoronal movement ^5^^,^^9^.

Regarding the transverse plane, more clockwise movement has been described, within a range of less than 0.5 mm, being mainly observed in teeth of the first and fourth quadrants and more pronounced in the maxilla than in the mandible ^5^. It has been reported that the lower canines usually change torque, presenting a change of 70° towards the buccal with the apex shorter and totally exposed, while different results have been described in the lateral incisors, which can move towards the vestibular or the lingual ^22^.

Usually the movements that occur with a fixed retainer involve changes in torques of two adjacent incisors known as the X effect, opposite inclinations of contralateral canines which is the rotational effect, and an increase in buccal and lingual inclination in individual canines ^5^^,^^11^^,^^22^. These terms were used by Kucera *et al*. ^23^ who indicated that the X effect is the change in torque of two adjacent teeth, indistinctly in upper or lower incisors, which may be accompanied by a rotation of the entire anterior segment and occlusal plane canting (rotational effect) in which small spaces develop between the teeth over time ^24^.

The X-effect pattern usually affects mandibular incisors, but can also affect adjacent teeth, leading to exposure of the apex root. Similar to the rotating effect that can occur in both canines and incisors a tilting towards lingual can be observed ^23^. In other study, lingual tilt, mesiorotation and distoangulation were detected, all of which were clearly observed from the first month of post-orthodontic control ^25^.

Translational or rotational movements are produced in the six anterior maxillary or mandibular teeth, and can be asymmetrical movements due to different causes, related to either materials or adhesion, which induce changes causing loss of dental alignment ^5^^,^^9^. Various software has been used to measure these movements such as the angulation from the vestibular axis. The inclination measured from its axis and the rotation have been analyzed by measuring the change in the rotational angle around the axial axis of the tooth which resulted in the previously mentioned effects ^26^.

Lastly, it is important to note that unwanted movement can occur long after the fixed retainer has been placed. Complications have been reported 2 to 4 years after the retainer placement ^22^^,^^27^. This is because patients with retainers should be regularly evaluated in the first two years during the initial retention periods since early detection of any complication such as dental movement is important in order to carry out timely and adequate corrective measures ^24^.

### Types of fixed retainer materials and their potential for relapse

In the bibliographic search, 36 articles related to the types of fixed retainers materials and their relapse potential were found- Fifteen of these articles were included in this review because they were considered to provide the greatest contribution to this topic and presented the greatest methodological rigor.

There is currently no consensus among orthodontists regarding the ideal type of wire for the use of fixed retainers ^28^^-^^30^. The most frequently used materials are multi-strand flexible stainless steel wires, which are attached to all anterior mandibular teeth and thick single-strand stainless steel, cobalt-chromium, or titanium-molybdenum wires bonded only to the canines ^31^. Multi-strand stainless steel wires (MSW) have been used since the early 1980s because of their flexibility, which allows a minimum amount of physiological movement between adjacent teeth, which in turn reduces the risk of breakage and, in addition, its textured and grooved surface allows ideal retention for composite resins ^18^.

Thin flexible multi-strand wire retentions are available in different diameters, cross-sections and internal organization of the strands. Thin, flexible wire diameters range from 0.0155" to 0.0215" for the round cross section or 0.016" x 0.022" for the rectangular cross section. The number of strands ranges from 3 to 8 and these can be arranged and manufactured in different patterns, which may be twisted, braided or coaxial ^31^. However, flexible 3-strand wires have been questioned in the literature for their stability and torsional stiffness, and the use of thicker 5-strand wires or even rigid stainless steel rectangular wires has been suggested to avoid unexpected changes after treatment ^11^.

Fiber-reinforced composites (FRC) are used in orthodontics as an esthetic, metal-free alternative to fixed retainers. Their advantages include biocompatibility, especially for patients who are allergic to nickel. In addition, they are lighter and easily contoured onto lingual tooth surfaces ^14^. However, FRC retainers are more sensitive to operator skill, and if improper bonding technique is used, the possibility of failure events is much greater ^15^.

Nagani and Ahmed14 compared the relapse trend of FRC retainers with MSW retainers at five different intervals every three months after cementation using Little's irregularity index. They found that the tendency to relapse was significantly greater in MSW than in FRC retainers at all time intervals, with a mean difference of 0.5 mm at 9 and 12 months after retainer insertion. It is mentioned that this result may be due to the greater rigidity of the FRC and to the splinting effect that causes an increase in tension levels and a reduction of relapse. In contrast, MSW retainers are so flexible that they allow more movement of the teeth, more frequently resulting in relapse. Another possibility for tooth movement with MSW retainers is a change in the mechanical properties of the wire, which can occur when the end is cut and the end tends to separate as it untwists causing a discrepancy in torque between adjacent teeth ^4^.

On the other hand, another study evaluated bond failure between MSW wire and FRC and concluded that MSW is a superior option for fixed lingual retention in the lower arch, since it presents less bond failure compared to the FRC retainer ^32^.

High-precision retention systems manufactured by computer-aided design and computer-aided manufacturing (CAD/CAM) software programs and made of nickel titanium or zirconium appear to be a promising alternative, considering that a perfect fit could prevent unwanted deformation during bonding.^26^ Although two recent studies found that CAD/CAM fabricated nitinol retainers do not provide superior stability or less gingival influence compared to classic braided stainless steel wire retainers ^15^^,^^33^.

Several studies have evaluated the failure rate of fixed retainers and its relationship with the different types of wires used. Although the current literature describes a failure rate ranging from 7.3% to 50%, the available scientific evidence does not support superior quality of any type of wire in terms of failure rate or alignment stability ^3^^,^^6^^,^^15^^,^^34^. Some studies show that flexible coiled wires are more likely to debond and that 0.027" titanium molybdenum alloy (TMA) round wire and 0.016"× 0.022" stainless steel stranded wires do not tend to break during 15 years of follow-up. However, stainless steel wires are more prone to detach than TMAs. It is worth mentioning that the failures tend to occur mostly within 2 years after retainer placement, mainly within the first 3 to 6 months at the time of the first check-up ^15^. Therefore, frequent control of retention status is essential in the first 6 months after retainer placement.

## CONCLUSIONS

• Post-orthodontic treatment relapse is the result of a regression towards the original malocclusion. However, changes in the position of the teeth can also occur, which are considered as unwanted movements and have a multifactorial origin. For this reason, it is necessary to treat orthodontic patients as if they have a high potential for relapse, and prior to receiving orthodontic treatment they should be informed about possible relapse and the methods currently used to prevent it.
• Unwanted or unexpected movements can occur with any fixed retainer due to causes that include the material used and the adhesion performed and are observed in the three spatial planes. It is important to note these movements over time by carrying out periodic controls, especially in the two first years of retention.
• Currently, in relation to alignment stability there is no consensus in the literature about the best type of orthodontic wire to use for fixed retention. Therefore, frequent controls are recommended after the retention has been cemented, especially within the first six months post-retention.

## Financing Sources:

Self-financed
